# THE ROLE OF FAMILY AND SOCIAL INTERACTIONS IN THE ASSOCIATION OF FAMILY BEREAVEMENT AND PSYCHOLOGICAL WELL-BEING

**DOI:** 10.1093/geroni/igae098.3065

**Published:** 2024-12-31

**Authors:** Nahyun Kim, Haeun Shin, Kyungmin Kim

**Affiliations:** Seoul National University, Seoul, Republic of Korea; Seoul National University, Seoul, Republic of Korea; Seoul National University, Seoul, Republic of Korea

## Abstract

Older adults may experience the death of family members more frequently than ones in earlier stages of life, and such family losses can affect psychological well-being in late life. After experiencing family losses, social interactions with remaining family members and acquaintances can be important resources for psychological adjustment. Bereaved individuals may interact more frequently with remaining family members following family deaths. Some people may be more socially engaged to cope with the negative effects of family losses, while others may reduce their social activities. This study examined the mediating role of intergenerational contact and social participation in the association between the total number of family deaths (occurred in the past 10 years) and psychological well-being (i.e., life satisfaction and depressive symptoms). We analyzed a sample of 2,279 respondents (aged 65–80) from the 2020 Korean Longitudinal Study of Ageing. The results indicated that there was no direct association of the number of family deaths with life satisfaction and depressive symptoms. However, we found one significant indirect path; experiencing more family deaths was linked to more frequent physical contact with non-coresident children, which in turn, led to higher levels of life satisfaction. Yet, the frequency of participation in social meetings was not significant as a mediator in the link between the number of family deaths and psychological well-being. Our findings suggest the importance of considering multiple family losses in late life and investigating underlying mechanisms linking accumulative life stressors to psychological adjustment among older adults.

